# The exploration-exploitation trade-off in a foraging task is affected by mood-related arousal and valence

**DOI:** 10.3758/s13415-021-00917-6

**Published:** 2021-06-04

**Authors:** Roel van Dooren, Roy de Kleijn, Bernhard Hommel, Zsuzsika Sjoerds

**Affiliations:** Cognitive Psychology Unit, Institute of Psychology & Leiden Institute for Brain and Cognition, Leiden University, Wassenaarseweg 52, 2333 AK Leiden, The Netherlands

**Keywords:** Exploration, Exploitation, Flexibility, Persistence, Mood, Foraging

## Abstract

The exploration-exploitation trade-off shows conceptual, functional, and neural analogies with the persistence-flexibility trade-off. We investigated whether mood, which is known to modulate the persistence-flexibility balance, would similarly affect the exploration-exploitation trade-off in a foraging task. More specifically, we tested whether interindividual differences in foraging behavior can be predicted by mood-related arousal and valence. In 119 participants, we assessed mood-related interindividual differences in exploration-exploitation using a foraging task that included minimal task constraints to reduce paradigm-induced biases of individual control tendencies. We adopted the marginal value theorem as a model-based analysis approach, which approximates optimal foraging behavior by tackling the patch-leaving problem. To assess influences of mood on foraging, participants underwent either a positive or negative mood induction. Throughout the experiment, we assessed arousal and valence levels as predictors for explorative/exploitative behavior. Our mood manipulation affected participants' arousal and valence ratings as expected. Moreover, mood-related arousal was found to predict exploration while valence predicted exploitation, which only partly matched our expectations and thereby the proposed conceptual overlap with flexibility and persistence, respectively. The current study provides a first insight into how processes related to arousal and valence differentially modulate foraging behavior. Our results imply that the relationship between exploration-exploitation and flexibility-persistence is more complicated than the semantic overlap between these terms might suggest, thereby calling for further research on the functional, neural, and neurochemical underpinnings of both trade-offs.

## Introduction

The adaptivity of human behavior often is attributed to cognitive control, the ability to tailor basic cognitive abilities to the task and situation at hand. Adaptive control often requires the solution of control dilemmas (Goschke, 2003), of which several have received substantial attention in the literature. Among those are the exploration-exploitation dilemma—addressing the question whether one should continue using the currently available knowledge or seek for new information—and the stability/persistence-flexibility dilemma (or persistence-flexibility dilemma for short)—tackling the question whether one should stick to one’s current goal or trade it for another, more tempting or easier to achieve alternative. In the present study, we were interested in possible functional commonalities between these control trade-offs.

We were motivated by the observation that the exploration-exploitation and persistence-flexibility dilemmas share many characteristics with respect to their descriptions in the literature. For one, the theoretical concepts of exploration and flexibility both imply that the present control settings need to be overruled and that the competitive strength of alternatives is increased, whereas the concepts of exploitation and persistence both refer to the continued maintenance of the current control settings in the more or less active suppression of alternative settings. Indeed, previous work within the domain of cognitive psychology has not drawn a sharp line between exploration-exploitation on the one hand and flexibility-persistence on the other (Hills, 2006; Hills & Dukas, 2012; Hommel & Colzato, 2017; Marković et al., 2019). For instance, and among others, Hills et al. (2008, 2010) have illustrated that such theoretical overlap is not far-fetched, because abstract conceptual search can be primed by inducing explorative or exploitative foraging strategies, with high levels of exploration (vs. exploitation) boosting flexible (vs. persistent) cognitive search patterns.

Along similar lines, even though both trade-offs have been addressed from different computational accounts, functional overlap between these control dilemmas can be inferred. For instance, the exploration-exploitation dilemma is often discussed in the context of (food) foraging (Wolfe, 2013). Exploiting an area that provides known and preferred food or rewards is typically energy-saving, as prediction-error signals decrease with increased expectation-outcome matches. However, at a particular point in time, provided that the availability of food or reward is subject to time and/or consumption, it might become more efficient to invest time and effort into moving to another area and start exploring for new resources (however, see Riefer et al., 2017). Hence, to remain efficient and keep the energy cost optimally low, foraging calls for an adaptive strategy in balancing the exploration-exploitation trade-off. In the same vein, an adaptive strategy can be distilled from the conceptualization of the persistence-flexibility trade-off. Specifically, having selected and persistently implemented a goal, task, or action is not sufficient for guaranteeing smooth performance, and might be increasingly difficult or mentally costly to maintain, especially in the presence of external stimuli or internal thoughts that promote other, competing goals, tasks, or actions (Atkinson & Birch, 1970). Protecting the current action goal against such interference requires active goal shielding, which would need to result in the suppression of alternative goals and respective response tendencies (Dreisbach & Goschke, 2004). However, in changing environments it may not always be the most optimal strategy to persist in the current action goal. Under such conditions, it would be adaptive to open up for other options that might outcompete the present goal—to become more flexible that is, reflected in a weak influence of the current goal on selection and weak competition between alternative codes (Hommel, 2015; Hommel & Colzato, 2017). Therefore, the exploration-exploitation and persistence-flexibility dilemma’s might be comparable in the fact that adaptive control is required to find an appropriate balance between choosing between familiar, but sometimes suboptimal alternatives and novel, often risky alternatives (Cohen et al., 2007).

Finally, in addition to these conceptual, and perhaps even functional overlaps, commonalities regarding the assumed neural mechanisms underlying the solution of the two dilemmas could similarly be distilled (Hills, 2006). For instance, tonic levels of norepinephrine (NE), reflecting more enduring and less discriminative neural responsiveness (Aston-Jones & Cohen, 2005), have been claimed to reduce the exploration-exploitation dilemma by promoting exploratory behavior (Jepma & Nieuwenhuis, 2011). Analogously, tonic NE levels have been suggested to play a role for the persistence-flexibility trade-off by biasing perception and memory in favor of salient, high priority representations (possibly reflecting increased persistence) at the expense of lower priority representations (possibly reflecting reduced flexibility; Mather et al., 2016). Similarly, dopamine (DA) has been suggested to play a central role in resolving both the exploration-exploitation trade-off (Cohen et al., 2007; Frank et al., 2009; Frank & Fossella, 2011; Hills, 2006; Hills et al., 2010; Kayser et al., 2015) as well as the persistence-flexibility dilemma (Cools & d’Esposito, 2011; Dreisbach et al., 2005; Dreisbach & Goschke, 2004; Durstewitz & Seamans, 2008; Hommel & Colzato, 2017).

Altogether, these commonalities seem to suggest that the semantic, functional, and perhaps even neural mechanisms responsible for finding the best balance between exploration and exploitation might, at least to some extent, overlap with those responsible for balancing flexibility and persistence. We admit that by theoretically equating these concepts, we do not do full justice to the tremendous amount of work that has been dedicated to the systematic study of both control dilemmas. For instance, research within the domain of decision making has shown that the operationalization of exploration and exploitation is dependent on the field of study, especially when it comes to its application in foraging behavior. While models of the explore-exploit dilemma differentiate between explicitly seeking out information versus acting on known rewards (Cohen et al., 2007), models of foraging center on probabilistically estimating average- and current reward rates (Charnov, 1976). However, even though these conceptualizations are often conflated (Todd & Hills, 2020), this does not render the core premise any less valid. In both instances, the goal of the organism is to maximize reward. In any case, we point out that a full-fledged discussion on these exact operationalizations, as well as a formalization of consensual definitions, is beyond the scope of this study and admit that our understanding of the two trade-offs as sharing semantic, functional, and neural commonalities is not necessarily consistent with all available explicit and implicit definitions of exploration/exploitation and persistence/flexibility. Taking these caveats into consideration, we speculated that factors that are known to have a systematic impact on people’s tendency to engage in more flexible or more persistent behavior should have a similar effect on the probability to show more explorative or more exploitative behavior.

Here we focused on a factor that has successfully been used to bias behavior away from persistence towards greater flexibility: mood. While the personal experience of mood is unlikely to have a causal impact on cognitive control processes (Hommel, 2019), the neurochemistry underlying mood changes has been found to systematically covary with changes in control policies. Moreover, induced positive mood, compared with negative or neutral mood, has been shown to promote cognitive flexibility and reduce persistence in a cognitive set-switching task (Dreisbach & Goschke, 2004), to reduce goal refreshing after conflict trials (Van Steenbergen et al., 2010), to promote divergent thinking in a creativity task (Akbari Chermahini & Hommel, 2012), and to reduce proactive control in a working memory updating task (Fröber & Dreisbach, 2014). Provided that all these observations can be validly taken to indicate that positive mood tends to shift control away from persistence and towards greater flexibility (Dreisbach & Goschke, 2004; Goschke & Bolte, 2014; Hommel & Colzato, 2017), we hypothesized that—if exploration-exploitation would show overlap conceptually and mechanistically with flexibility-persistence—inducing positive mood before presenting a task that is sensitive to exploration-exploitation tendencies should generate a stronger bias towards exploration. Such expectations are in line with observations as reported within the domain of affect and decision making, in which positive-going mood states have been shown to favor exploratory behavior (Vinckier et al., 2018), while negative-going mood states potentially result in overexploitation (Lenow et al., 2017).

Provided that mood induction is known to affect both perceived arousal and perceived valence measures, we also were interested to see whether these measures would differentially affect possible biases in exploration/exploitation, especially because arousal and valence have received a considerably different emphasis within accounts on the exploration-exploitation trade-off compared with literature on the persistence-flexibility balance. Specifically, according to Cohen et al. (2007), NE, which is assumed to be associated with mood-related arousal (Terbeck et al., 2016), plays an important role in regulating exploration and exploitation. In contrast, in persistence-flexibility accounts, valence is often explicitly or implicitly assumed to be the key player (Van Steenbergen et al., 2010).

Taken altogether, the main goal of the present study was to test whether and how the mood-related regulation of the exploration-exploitation balance relates to the regulation of the persistence-flexibility trade-off. To this end, we designed a foraging task that was adopted from Hills et al. (2008, 2010), who originally had used it to induce explorative and exploitative foraging strategies. Given that we did not intend to *induce or promote* a particular control mode, but rather to assess the way our mood induction manipulation would shift the relative emphasis on exploration and exploitation, we adjusted the task to provide participants the freedom to explore or exploit the task environment following their individual tendencies instead of specific task-conditions. We compared performance in this foraging task pre-mood induction with performance after positive versus negative mood induction, which we assumed to modulate the trade-off between exploration and exploitation the same way as it has been shown to modulate the balance between persistence and flexibility.

In particular, we tested the following hypotheses: First, and foremost, we expected mood induction to affect the exploration-exploitation trade-off. Second, given that positive mood has been found to promote flexible task performance (Dreisbach & Goschke, 2004; Fröber & Dreisbach, 2014; Van Steenbergen et al., 2010), we expected a positive-going mood to promote exploration, whereas a negative-going mood should promote exploitation. Finally, given the differential roles of arousal and valence in modulating the exploration-exploitation versus persistence-flexibility trade-offs, we tested whether arousal, valence or both measures would orthogonally modulate foraging behavior.

## Methods

### Participants

A total of 119 participants (78 females; *M*_*age*_ = 21.83, *SD*_*age*_ = 1.97) was recruited via the Research Participation System of the Leiden Institute of Psychology (Leiden University, Netherlands). Participants were in good mental and physical health and did not have a history of psychiatric or neurological conditions (as assessed by the Mini International Neuropsychiatric Interview; Sheehan et al., 1998), had normal or corrected-to-normal eyesight and were between ages 18 and 27 years. Participants provided written, informed consent and were naïve to the purpose of the experiment. An experimental session took approximately 45 minutes. In return for their participation, participants received either 2 course credits or €5. The study protocol was approved by the local ethics committee (Leiden University, Institute of Psychological Research; CEP19-0221/116).

Due to the novelty of our approach, it was challenging to properly estimate the best sample size before data collection. We did consult literature incorporating similar mood induction procedures in combination with cognitive control assessment (Jefferies et al., 2008; Van Steenbergen et al., 2010), which informed us that such manipulations typically include 20-25 participants per condition. However, such small samples sizes likely result in low statistical power. Indeed, we calculated that with an effect size of η_p_^2^ = 0.05 (Van Steenbergen et al., 2010, personal correspondence) a power of <0.5 would be obtained. Therefore, based on the effect sizes estimated from Van Steenbergen et al. (2010), we performed *a priori* power calculations in GPower (Erdfelder et al., 1996) to estimate the minimum sample size for this study. Results indicated that a total sample size of 115-120 participants would be sufficient to detect such low effect sizes (i.e., η_p_^2^ = 0.05) with a power of 0.80, and an alpha level of 0.05 (one-sided). Additionally, we included Bayesian analysis methods, because such approaches allow for explicit testing of the null versus the alternative hypothesis and provide possibilities for the post-hoc assessment of statistical power. Notably, we omitted data of two participants, because these participants indicated that they did not understand the structure of the foraging task, and hence showed inactivity in the task for a substantial amount of time. Consequently, the sample size for our analyses was reduced to a total of 117 participants.

### Procedure

After arrival, participants were comfortably seated in front of a computer, at a distance of approximately 60 cm from the screen (AOC I2475PXQU monitor: 1920 × 1080, 23.8 inch). They read and signed the informed consent, and demographics were assessed through standardized questions*.* On-screen instructions guided them through a sequence of four tasks: (1) a practice navigation task, (2) a pre-mood induction run of the foraging task, (3) a mood induction procedure (excited vs. sad, counterbalanced between participants), and (4) a post-induction run of the foraging task. In order to dynamically capture changes in mood state, a 9 × 9 arousal by valence mood grid (Russell et al., 1989) was presented at multiple time points throughout the experiment. Upon completion of the experiment participants were debriefed and received their payment.

### Materials

#### Practice navigation task

Before the presentation of the experimental sequence, participants completed a navigation task to familiarize themselves with the joystick they would use within the foraging task runs. To this end, participants had to move a character (19 × 23 pixels) through a two-dimensional maze (600 × 600 pixels). The character’s speed (0–60 pixels per second) and angle (0°–359°) could be adjusted by means of the joystick. The goal was to successfully navigate the character to the exit of the maze. The trial was reinitialized if participants hit any of the maze’s walls. No time restrictions were set. Average duration spent on this practice task was 23.05 seconds (*SD* = 8.07 seconds).

#### Foraging task

The design of our foraging paradigm was inspired by the foraging task as described by Hills et al. (2008, 2010). However, while the original task by Hills and colleagues included two separate conditions aimed at *inducing* either exploitative or explorative foraging behaviour between-subjects (i.e., clumpy vs. diffuse resource distributions, respectively), the set-up of our task was aimed at *measuring* individual foraging tendencies within-subjects. Therefore, we designed a task with as few task constraints as possible to reduce paradigm-related impact, enabling us to measure individual’s, task-unconstrained tendency to explore versus exploit.

To effectively capture dynamic adjustments in foraging strategies, we provided participants with a “zoom-in to patch view/zoom-out to aerial view” feature, which allowed them to approach the foraging task as preferred. Specifically, this feature enabled participants to, at their own pace and as much as they preferred, freely explore the foraging environment in order to locate new resource patches, or return to already discovered resource patches. Moreover, it allowed participants to start exploiting previously unentered resource patches, or to start re-exploiting previously entered resource patches. Hence, we assume that the small adjustments we made with respect to the original task enable us to measure inter-individual differences in foraging strategies, with foraging behavior largely depending on individuals’ internal foraging preference or strategy, which one could continuously update (e.g., from exploration to exploitation), unconstrained by task conditions. The foraging paradigm was coded in Python 2.7.

Participants were instructed to find as many resources (i.e., berries) within a restricted period of 5 minutes. Resources were hidden in patches (i.e., bushes), which in turn were hidden in a game environment (600 × 600 pixels). Each trial comprised nine unique patches (radii of 57 pixels), each constituting 40 to-be-discovered resources. The patch-positions were randomly drawn from two uniform distributions (for the x- and y-coordinates, respectively), with the notable exceptions that (i) the minimum distance between patches was set to 9 pixels, (ii) patches were to be located at a minimum distance of 15 pixels from any of the environment’s borders, and (iii) the minimum distance between the center of the environment and a patch constituted at least 57 pixels. The distribution of the patches thereby conceptually mirrored the clumpy resource distribution as described by Hills et al. (2008, 2010).

**Fig. 1:**
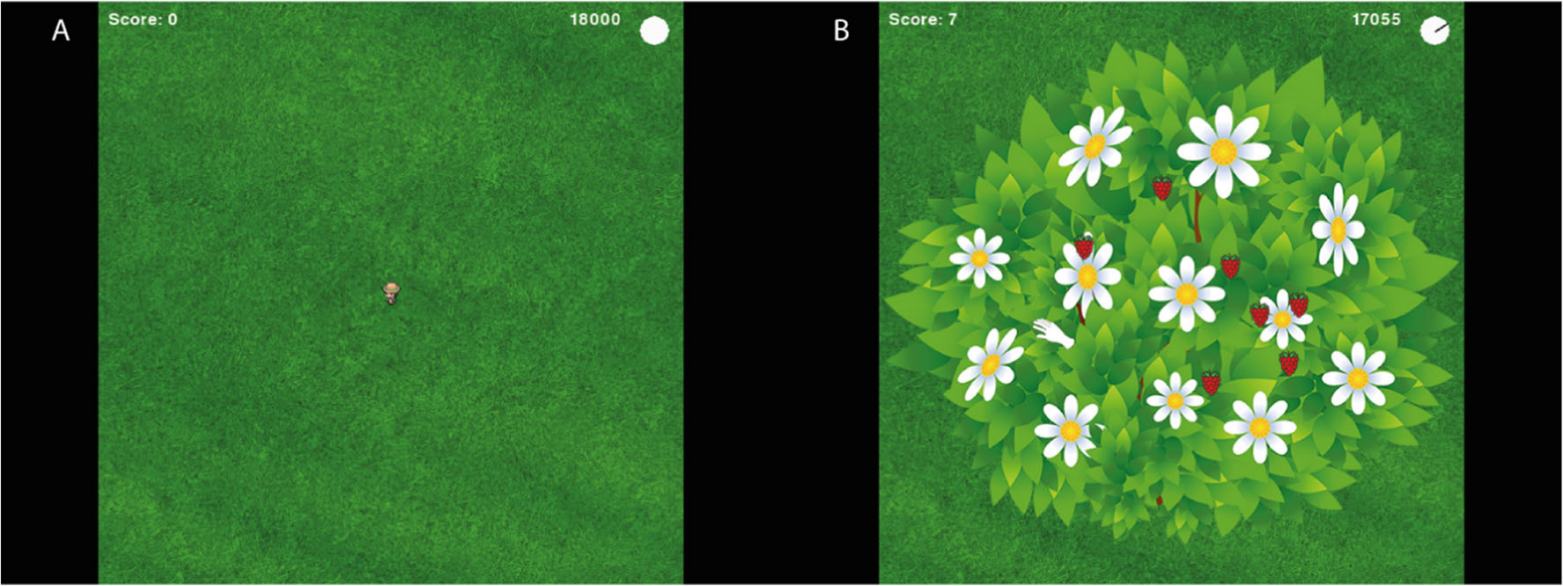
Visual representation of the two layers of the foraging task, displaying the aerial-view (**a**) and patch-view (**b**)

At the start of a trial, participants were presented with a game environment depicting this so-called aerial view (Fig. 1a). A character (19 × 23 pixels) was positioned in the center of the environment, facing a random location in space. The character could be moved by means of the joystick with a speed of 0-60 pixels per second and an angle of 0°–359°. If the character was navigated to a position where a patch was hidden, the part of the patch that was present within a 15-pixel radius of the character’s center location became visible. Once a patch was encountered, it could be entered by pressing a key on the back-side of the joystick. Upon patch entry, a new game environment (600 × 600 pixels) was rendered, which displayed a zoomed-in view of the entered patch (i.e., patch view; Fig. 1b). Within each patch, resources (15 × 15 pixels) were randomly distributed, with the precondition that resources were not allowed to be in a 30-pixel vicinity of any other resource. Hence, the distribution of resources within patches conceptually mirrored the diffuse resource distribution as described by Hills et al. (2008, 2010). The character was replaced by the image of a hand (49 × 44 pixels), which could, by means of the joystick, be moved with a speed of 0-90 pixels per second and an angle of 0°–359°. If the hand was navigated to a position where a resource was hidden (i.e., within a 30-pixel radius of the hand’s center location), the resource became visible. Each found resource, equaling 1 point, was displayed on screen as a red berry (33 × 33 pixels). Importantly, before the start of the foraging trial, participants were informed that resources could *only* be found once a patch was entered (i.e., in patch view), while new patches could *only* be discovered in aerial view. Moreover, they were informed that the three participants that, overall, collected most resources, were rewarded with an additional voucher of €15.

In line with the task design of Hills et al. (2008, 2010), a counter was presented in the top-right corner of the environment. The counter started at a value of 18,000 (i.e., the maximum speed in pixels per second within the aerial view multiplied by the 300-second duration of a foraging run) and decreased with a value of 60 units per second. Adjacent to this counter, a sweeping clock-hand was presented. Together, these tools informed participants that time was running out, without providing direct information on the actual time that remained. Finally, the total amount of resources found was continually updated and presented in the upper-left corner of the screen.

#### Marginal value theorem

To investigate participants’ general tendencies to explore/exploit in the foraging task, we adopted the marginal value theorem (MVT; Charnov, 1976), which aims to characterize and provide an approximation of optimal foraging behavior by tackling the patch-leaving problem.

The marginal value theorem posits that during foraging in a patchy environment, the amount of food gained for time *T* spent in a patch is *f(T)*, with *f(T)* rising from zero at a negatively accelerated rate to an asymptote representing the total amount of food present in a patch. As the first derivative of *f(T),* the current rate of food intake, approaches zero, an organism will not derive any benefit from staying in that particular patch as it will have exploited all its resources. When exactly should an organism move away from its current patch to the next? In the original case of multiple patch types, the average time *T* spent to use one patch is the travel time plus the time spent in the patch:

$$ T=t+{P}_A\cdotp {T}_A+{P}_B\cdotp {T}_B $$

and the average amount of food *E* taken from a patch is

$$ E={P}_A\cdotp f\left({T}_A\right)+{P}_B\cdotp f\left({T}_B\right) $$

in which *P*_*A*_ and *P*_*B*_ are the proportion of patches visited that are of type A or B. In the current paradigm, this can be simplified to

$$ T=t+{T}_P,E=f\left({T}_P\right) $$

in which *T*_*P*_ is the time spent in any patch. It follows then that E/T reflects the average rate of food intake, which an organism should try to maximize. To find this maximum, we can find the partial derivative of E/T using the quotient rule, setting

$$ \frac{\delta \left(E/T\right)}{\delta {T}_P}=0, $$

we then find that this is the case when

$$ \frac{\delta f\left({T}_P\right)}{\delta {T}_P}=E/T, $$

or, in other words, when the current food intake rate *f(T*_*P*_*)* is identical to the average food intake rate E/T. In the current paper, we will refer to these variables as the current reward rate CRR and the average reward rate ARR, respectively. Following MVT, exploitation should be preferred over exploration whenever the CRR is higher than the ARR. In other words, one should shift from an exploitative to a more exploratory foraging mode whenever the instantaneous reward rate becomes lower than the average expected reward rate. Because in the present paradigm rewards are received in a discrete instead of a continuous manner (i.e. the *actual* received reward is either 0 or 1), the CRR parameter was calculated over a short time interval.

#### Optimal window for calculating the CRR

**Fig. 2:**
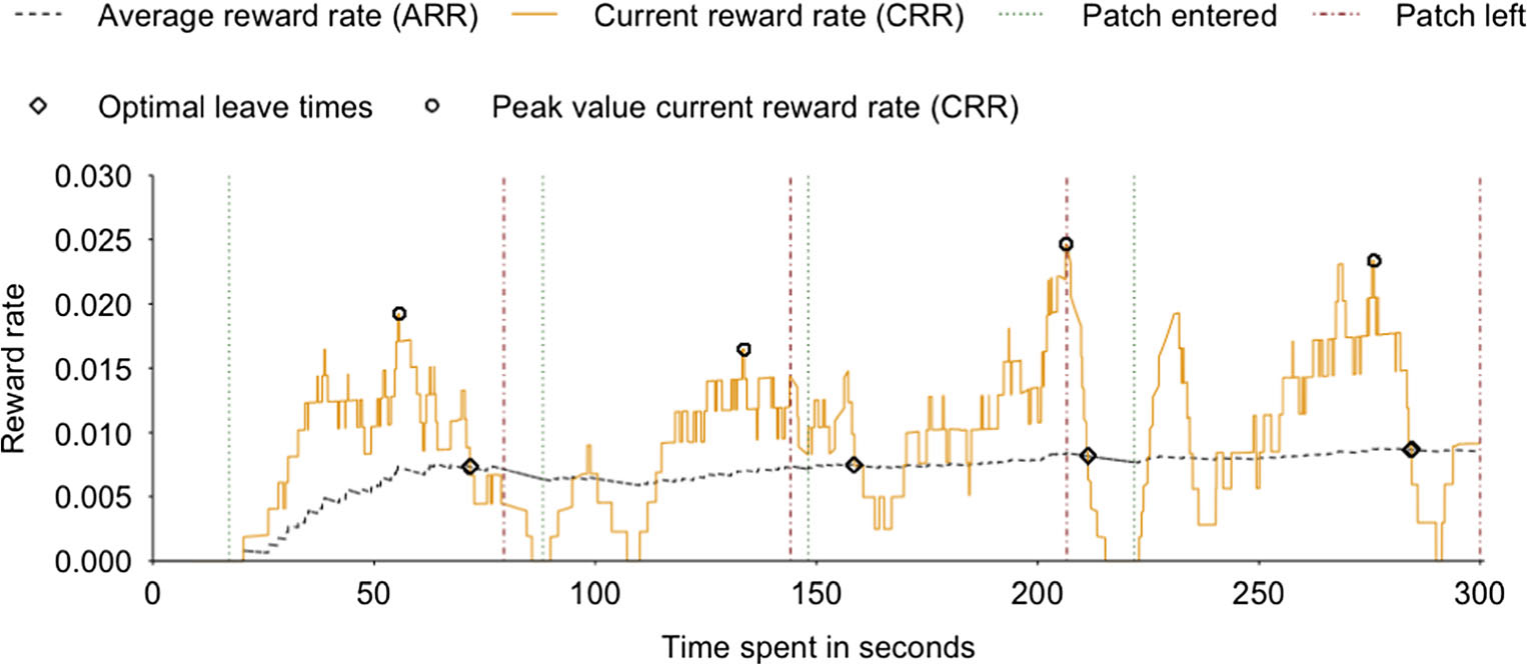
ARR/CRR fitting procedure for one specific participant. To approximate optimal foraging behavior (following MVT), we calculated, separately for each participant and each time point, the current reward rate (CRR) and average reward rate (ARR) of the environment. Instances where the CRR and ARR intersect after a peak in the CRR signal is detected (displayed as circles) reflect optimal leave times (OLTs; displayed as diamonds). These time points were compared with participants’ actual leave times (i.e., ALTs; vertical red lines), which defined the leave time difference (LTD) parameter

To determine an interval over which to calculate the CRR, we iteratively fitted the data of the first foraging run of each participant for multiple time windows (Supplementary Figure 1). Our fitting procedure extracted, separately for each participant and each patch visit, the interval for which, on average, the difference between the optimal leave time (OLT) and participants’ actual leave time (ALT) was closest to 0 (i.e., leave time difference; LTD). In Fig. 2, OLTs are reflected as moments where the CRR and ARR intersect after a peak in the CRR signal is detected.

By adopting this normalization step, deviations from 0 on the LTD parameter on the foraging run following the mood induction procedure would thereby likely reflect effects induced by the manipulation, with a negative LTD reflecting an emphasis on exploitation over exploration (OLT < ALT) and a positive LTD implying a more exploratory foraging mode (OLT > ALT). We acknowledge that this is only one method for determining what interval to use to determine the CRR, but considering we are interested in *changes* to foraging behavior the exact definition of CRR should not matter.

Within the first iteration, the LTD parameter was calculated for a total of 200 unique time windows (0–20 seconds in steps of 100 milliseconds). The analysis revealed that, on average, LTD was smallest for an interval of exactly 9.00 seconds (i.e., OLT - ALT = −0.024). To confirm that we extracted the most optimal time window over which to calculate the CRR, we repeated this procedure in a second iteration by estimating the LTD parameter for another 50 unique time windows (8.75–9.25 seconds in steps of 10 milliseconds). Once again, the analysis revealed that, on average, LTD was smallest for the 9.00-second time window. Importantly, a between-subjects ANOVA confirmed that, pre-mood induction, the LTD parameter for participants in the excited and sad mood induction conditions (see below) did not significantly differ (*F*(1, 115) = 0.30, *p* = 0.59, ηp^2^ < 0.01).

#### Mood induction

The mood induction protocol used music to manipulate mood, a procedure that has successfully been used before (Eich et al., 2007; Van Steenbergen et al., 2010). Our induction protocol incorporated two conditions to which participants were randomly assigned: a positive (excited; high arousal, high valence) or negative (sad; low arousal, low valence) induction condition (*n*_excited_ = 57, *n*_sad_ = 60). During the mood induction procedure, participants listened to classical music congruent with the mood condition whilst writing down a memory or hypothetical situation in which they have experienced or would hypothetically experience the intended mood of the condition. The design was a double-blind study design, in the way that participants were not informed beforehand that we tested two different mood conditions, and the experimenter did not know the condition participants were in, as the induction happened using headphones, and the written stories were not read by the experimenter. The mood induction phase lasted 10 minutes. Participants in the sad condition that indicated to still be in a negative mood during the debriefing were given the option to go through the positive mood induction.

#### Mood grid

Participants’ arousal and valence were assessed by means of a two-dimensional (9 cells × 9 cells) Likert scale (Russell et al., 1989). Participants were instructed to mark 1 of 81 unique cells in the matrix. From left to right and from bottom to top, arousal and valence were scored from *very sleepy* (−4) to *highly aroused* (+4) and from *very unpleasant* (−4) to *very pleasant* (+4), respectively. For example, if participants placed a cross in the upper-right corner of the grid, this would indicate feelings of excitement (high valence, high arousal). This relatively simple instrument has been found to have adequate reliability and validity (Russell et al., 1989). Participants were asked to fill out the mood grid a total of 6 times: before and after each of the foraging task runs (i.e., time points 1, 2, 5, and 6), and before and after the mood induction procedure (i.e., time points 3 and 4). The direct effect of the mood induction on mood was assessed by the arousal and valence ratings on the third and fourth mood grid.

### Statistical analyses

All analyses were performed in the analysis software R (R Core Team, 2019; Version 3.6.0), with the critical alpha value for significance being set to *p* = 0.05, while correcting for multiple comparisons. Bayesian analyses with an uninformative model prior were additionally conducted to assess the strength of observed results. These analyses were performed using the BayesFactor package (Morey & Rouder, 2018).

To assess whether mood ratings were significantly modulated by our mood induction manipulation, we subjected participants’ arousal and valence ratings to two separate repeated-measures ANOVAs (*ezANOVA* from the R package *ez*) with Condition (excited vs. sad) as a between-subject factor and Time point (1 vs. 2 vs. 3 vs. 4 vs. 5 vs. 6) as a within-subject factor. In case of a violation of the sphericity assumption, Greenhouse-Geisser corrected *df* and *p* values are reported and error-terms are provided. For those analyses for which we had specific expectations regarding the directions of effects, one-tailed *t*-tests were performed. Associated *df* values were corrected by means of Welch’s approximation in case of a violation of the assumption of homogeneity of variances, and *p* values were accordingly corrected for multiple comparisons (Holm, 1979). To check whether groups did not differ in mood before the mood induction manipulation (time points 1 through 3), each of the RM-ANOVAs was followed up with planned post-hoc t-tests.

To assess whether (changes in) arousal and valence modulated behavior in the foraging task, we conducted three hierarchical multiple linear regression analyses. For the first analysis, we calculated the mean ratings provided on the mood grids presented at time points 1 and 2 (i.e., pre-mood induction ratings) and used these continuous variables to predict LTD scores for the first foraging run, while the mean ratings at time points 5 and 6 (i.e., post-mood induction ratings) were extracted as a measure of these parameters for the analysis on the LTD data of the foraging run post-mood induction. In order to test whether changes in arousal and valence were related to adjustments in foraging behavior, we fitted the third multiple linear regression model using difference scores for each of the abovementioned parameters; delta arousal (post-mood induction arousal minus pre-mood induction arousal), delta valence (post-mood induction valence minus pre-mood induction valence), and delta LTD (post-mood induction LTD minus pre-mood induction LTD).

For each of the regression analyses, as a first step, a simple regression model was fitted. In a second step, these models were updated with the predictors’ higher-order interaction term. These hierarchical models were compared using the *anova* function from the R package *car*. In case a more complex model did not outperform a more parsimonious model, we selected the model with the lowest BIC score. All models reported below adhered to the assumptions of independent errors (*durbinWatsonTest* from the R package *car*), multicollinearity of residuals (*vif* from R package *car*), normality of residuals, linearity of residuals, and homoscedasticity (Tabachnick & Fidell, 2012).

Finally, for each of the foraging runs, we ran multiple hierarchical linear regression analyses to identify whether general foraging metrics were modulated by Condition (excited vs. sad) and arousal and valence scores. In particular, we focused on (1) the ratio of unique to total amount of patches visited, (2) the median time spent in patches, (3) the variability in patch leave times, (4) the mean travel time between patches, (5) the time elapsed before the first patch was entered, (6) the total area of the environment that was explored, and (7) the total amount of berries collected. Because these analyses are not within the main scope of the paper, these results can be consulted within the Supplementary Material.

## Results

### Arousal ratings

**Fig. 3:**
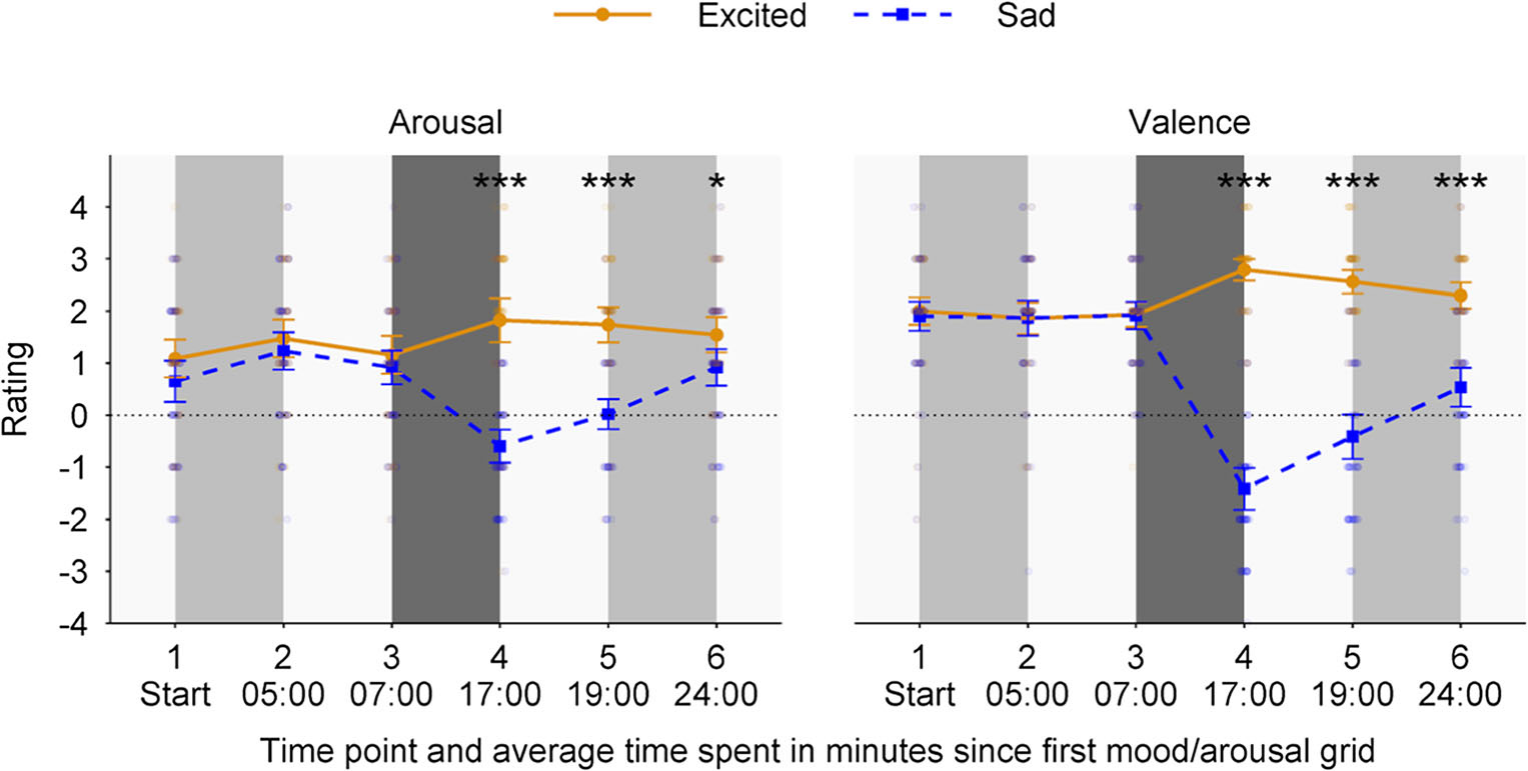
Self-reported arousal and valence ratings before and after each of the foraging runs (time points 1, 2, 5 and 6), and before and after the mood induction procedure (time points 3 and 4). The light-grey boxes indicate the moments at which participants performed the pre-mood induction (i.e., first light-grey box) and post-mood induction foraging runs (i.e., second light-grey box). The dark-grey box indicates the moment where the mood induction procedure was presented. Each of these time points has additionally been labeled as the average amount of elapsed minutes since the presentation of the first mood grid (i.e., time point 1). Individual data points are reported. Error bars represent 95% between-subject CIs. *p < 0.05, ***p < 0.001, Holm-corrected for multiple comparisons

The RM-ANOVA on the arousal data with factors Condition (excited vs. sad) and Time point (1 vs. 2 vs. 3 vs. 4 vs. 5 vs. 6) showed a main effect of Condition (*F*(1, 115) = 26.51, *p* < 0.001, ηp^2^ = 0.19) and a main effect of Time point (*F*(3.57, 575) = 8.42, *p* < 0.001, ε = 0.71, ηp^2^ = 0.07) (Supplementary Table 2). Overall, arousal ratings were higher for participants in the excited condition (*M* = 1.47, *SD* = 1.40) compared with participants in the sad condition (*M* = 0.52, *SD* = 1.46, see Supplementary Figures 2a-f for the associated 2D-heatmaps). Moreover, and as expected, a significant Condition × Time point interaction was observed (*F*(3.57, 575) = 24.07, *p* < 0.001, ε = 0.71, ηp^2^ = 0.17, Fig. 3). Along similar lines, the Bayesian RM-ANOVA showed strong evidence in favor of the alternative hypothesis (BF_10_ = 1.51×10^27^). More precisely, the estimated Bayes factor suggests that the data are 1.51×10^27^ times more likely under the alternative hypothesis—namely that the factors Condition and Time point affect arousal ratings—compared with the null hypothesis.

Post-hoc *t*-tests (Holm-corrected) revealed that arousal ratings for the excited and sad conditions did not significantly differ preceding the mood induction manipulation (time points 1 through 3, |*t|*s < 1.62, *p*s_*corr*_ > 0.16, *d*s < 0.30, 0.3 < BFs_01_ < 3). In contrast, for time points 4, 5, and 6, arousal ratings were significantly larger in the excited condition compared with the sad condition (|*t|*s > 2.57, *p*s_*corr*_ < 0.024, *d*s > 0.47, BFs_10_ > 7.25; Supplementary Table 4).

Finally, one-tailed paired-sample *t*-tests on the ratings provided at time points 3 and 4 confirmed that arousal ratings significantly increased for participants in the excited condition (|*t|*(56) = 2.85, *p* = 0.003, *d* = 0.45, 95% confidence interval [CI] [0.07, 0.82], BF_10_ = 5.48), whereas these ratings significantly decreased for participants in the sad condition (|*t|*(59) = 8.01, *p* < 0.001, *d* = −1.22, 95% CI [−0.82, −1.61], BF_10_ = 1.83×10^8^).

### Valence ratings

The RM-ANOVA on the valence data with factors Condition (excited vs. sad) and Time point (1 vs. 2 vs. 3 vs. 4 vs. 5 vs. 6) showed a main effect of Condition (*F*(1, 115) = 94.20, *p* < 0.001, ηp^2^ = 0.45) and a main effect of Time point (*F*(3.73, 575) = 39.25, *p* < 0.001, ε = 0.75, ηp^2^ = 0.25) (Supplementary Table 3). Overall, valence ratings were higher for participants in the excited condition (*M* = 2.24, *SD* = 0.99) compared with participants in the sad condition (*M* = 0.73, *SD* = 1.87, Supplementary Figures 2a-f for the associated 2D-heatmaps). Moreover, the analysis showed a significant Condition × Time point interaction (*F*(3.73, 575) = 115.22, *p* < 0.001, ε = 0.75, ηp^2^ = 0.50; Fig. 3). Along similar lines, the Bayesian RM-ANOVA showed strong evidence in favor of the alternative hypothesis (BF_10_ = 8.89×10^110^). The estimated Bayes factor suggests that the data are 8.89×10^110^ times more likely under the alternative hypothesis—namely that the factors Condition and Time point affect valence ratings—compared with the null hypothesis.

Post-hoc *t*-tests (Holm-corrected) revealed that valence ratings for the excited and sad conditions did not significantly differ preceding the mood induction manipulation (time points 1 through 3, |*t|*s < 0.52, *p*s_*corr*_ > 0.91, *d*s < 0.10, BFs_01_ > 3.28). In contrast, for time points 4, 5, and 6, valence ratings were significantly larger in the excited condition compared with the sad condition (|*t|*s > 7.86, *p*s_*corr*_ < 0.001, *d*s > 1.44, BFs_10_ > 3.82×10^9^; Supplementary Table 4).

Finally, one-tailed, paired-sample *t*-tests on the ratings provided at time points 3 and 4 confirmed that valence ratings significantly increased for participants in the excited condition (|*t|*(56) = 7.42, *p* < 0.001, *d* = 1.04, 95% CI [0.64, 1.43], BF_10_ = 1.54×10^7^), whereas these ratings significantly decreased for participants in the sad condition (|*t|*(59) = 15.42, *p* < 0.001, *d* = −2.53, 95% CI [−2.04, −3.01], BF_10_ = 1.88×10^19^).

### Validity of condition classifications

Interestingly, as can be distilled from Fig. 3, arousal and valence levels were fairly high for participants in the sad mood condition, both before and after mood induction. Specifically, although ratings of most participants in the excited mood condition fell into the expected upper half of the scores, indicating high arousal and valence (50/57 participants), a dissimilar pattern was observed for participants in the sad mood condition, as most participants still scored in the upper half of the grid, indicating high arousal and valence, and only a small part (14/60 participants) scored relatively low on these measures. Put differently, although our manipulation was successful in inducing variability in mood between groups, participants’ arousal and valence ratings did not necessarily conform to the pattern of ratings as expected from the predefined classifications (Van Steenbergen et al., 2010). Because we were interested in interindividual adjustments of control states as induced by our mood manipulation, we treated the self-reported arousal and valence ratings as continuous predictors within all subsequent analyses, rather than comparing discrete groups based on mood induction conditions. However, the interested reader can consult the Supplementary Material for all analyses incorporating the factor Condition. It is worth mentioning that, irrespective of whether the factor Condition was added to the models, the below described effects of interest remain unaltered.

### Foraging task metrics as predicted by mood

Three hierarchical multiple linear regression analyses were conducted to reveal whether (changes in) arousal and/or valence modulated participants’ LTD scores. Before model specification, four participants were removed, as their LTD value on either the pre-mood induction or post-mood induction foraging run exceeded the interquartile range by factor 2.2. Even though the below reported results did not change when including these LTD outliers, we nevertheless decided to exclude these observations as such omissions eliminated violations of model assumptions. Importantly, for each of the analyses reported below, ANOVAs revealed that updating the models did not significantly increase the model fits (all *F*s(1, 109) < 3.88, all *p*s > 0.051). All below-reported *p* values have been multiplied by a factor 3 to correct statistically for the total amount of performed regression analyses.

The regression analysis on the relationship between LTD and arousal and valence ratings pre-mood induction did not reveal any significant association (*F*(2, 110) = 3.05, *p*_*corr*_ = 0.15, *R*^*2*^ = 0.04; β = −0.14, 95% CI [−0.33, 0.04], |*t|*(110) = 1.51, *p*_*corr*_ = 0.39; β = −0.16, 95% CI [−0.34, 0.03], |*t|*(110) = 1.67, *p*_*corr*_ = 0.30, for arousal and valence ratings, respectively). The Bayesian linear regression analysis did not reveal sufficient evidence (0.3<BF<3) in favor of the null nor the alternative hypothesis (BF_10_ = 0.80).

Similarly, the second regression analysis did not reveal any relationship between the mood predictors and LTD post-mood induction (*F*(2, 110) = 0.41, *p*_*corr*_ = 1.0, *R*^*2*^ = −0.01; β = 0.00, 95% CI [−0.21, 0.21], |*t|*(110) = 0.01, *p*_*corr*_ = 1.0 and β = −0.09, 95% CI [−0.30, 0.12], |*t|*(110) = 0.82, *p*_*corr*_ = 1.0 for arousal and valence ratings, respectively). The Bayesian linear regression analysis showed strong evidence (BF_01_ = 11.79) in favor of the null hypothesis. Specifically, the estimated Bayes factor suggested that the data were 11.79 times more likely under the hypothesis that post-mood induction LTD and arousal and valence ratings post-mood induction were not related.

**Fig. 4:**
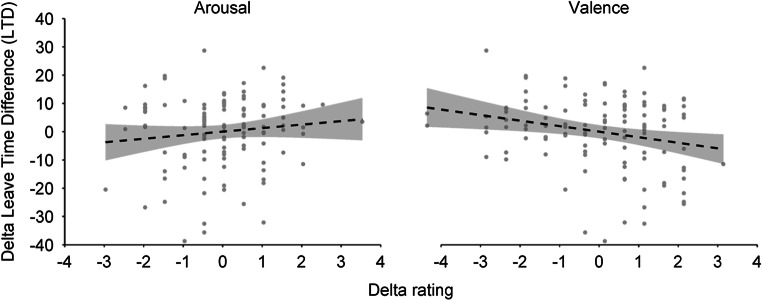
Delta arousal and valence ratings (post-mood induction ratings minus pre-mood induction ratings) predict delta leave time differences scores (post-mood induction LTD minus pre-mood induction LTD). Specifically, while increases in arousal are associated with increases in exploratory behavior, increases in valence are correlated with increases in exploitative behavior. Individual data points are reported. The band-widths represent 95% between-subject CIs

Finally, and interestingly enough, the third regression analysis revealed that delta LTD scores were significantly predicted by both delta arousal and delta valence ratings (*F*(2, 110) = 6.79, *p*_*corr*_ = 0.005, *R*^*2*^ = 0.09). Specifically, while an increase in arousal was associated with an increase in exploratory foraging behavior (β = 0.24, 95% CI [0.05, 0.44], |*t|*(110) = 2.51, *p*_*corr*_ = 0.041), an increase in valence was associated with an increase in exploitative behavior (β = −0.33, 95% CI [−0.53, −0.14], |*t|*(110) = 3.45, *p*_*corr*_ = 0.002; Fig. 4). Importantly, the Bayesian linear regression analysis showed strong evidence in favor of the alternative hypothesis (BF_10_ = 17.65). More precisely, the estimated Bayes factor suggested that the data were 17.65 times more likely under the alternative hypothesis—namely the hypothesis that changes in arousal and/or valence significantly correlated with adjustments in foraging behavior—compared with the null hypothesis.

## Discussion

Our main research goal was to study whether and how the regulation of exploration and exploitation relates to the regulation of the persistence-flexibility trade-off. To fill this gap, we induced, between-participants, two types of mood that are assumed to differ in arousal and valence (i.e., excited vs. sad), and subsequently tested whether these mood states differentially affected individual tendencies towards exploration/exploration as measured by means of a foraging task. As previously alluded to, our research aim can be divided into three questions, which we will address in turn.

The answer to the first question, whether changes in arousal and/or valence systematically affect exploration and exploitation, is clearly yes: not only did we manage to successfully induce the expected mood changes in our participants, but we also were able to show that these changes had an effect on participants’ tendency to explore/exploit in the foraging task. Specifically, adjustments in both arousal and valence were associated with changes in foraging behavior: while increases in arousal significantly correlated with increased levels of exploration, increases in valence substantially increased exploitative foraging behavior. From a computational perspective, the induction of a positive or negative mood could modulate either information integration or the decision mechanism. There is evidence from other domains to suggest that primacy and recency effects are affected by mood (see Forgas, 2011 for a social psychology approach), which may cause the difference in foraging behavior. However, such a conclusion seems problematic, as it can be argued that exploratory behavior from this point of view could be induced by an overestimation of the average reward rate or an underestimation of the current reward rate. As the current study found that an increase in arousal is associated with more explorative behavior, this would suggest that an increase in arousal leads to an overestimation of the average reward rate or an underestimation of the current reward rate. This suggests that a recency effect may be at play, as the average reward rate increases slowly overall starting the integration process at zero, while the literature suggests that increased arousal is associated with increased primacy effects (Cahilla & Alkireb, 2003). However, it is unclear if this translates to the current paradigm. Therefore, these observations only partly matched our initial expectation that positive-going mood states would, for both arousal and valence dimensions, promote exploration and negative-going mood would induce exploitation.

As a consequence thereof, the second question, asking whether possible effects on exploration/exploitation are conceptually similar to the effects one typically observes when regulating the persistence-flexibility balance by means of a mood induction procedure, can be denied: while the semantics and functional descriptions of the conceptual pairs suggest a strong commonality and possible overlap between exploration and flexibility on the one hand, and between exploitation and persistence on the other (Cohen et al., 2007; Hills, 2006), positive-going mood did not result in more exploration, but in more exploitation. This stands in contrast to the semantic and theoretical considerations that have previously been proposed (Hills et al., 2008, 2010; Hommel & Colzato, 2017) and suggests that flexibility cannot simply be equated with exploration and persistence not with exploitation, at least not with respect to the behavioral measures as included within this particular study design.

The lack of such a conceptual overlap directly taps into the third question, namely whether effects reflect the impact of arousal, a key player in exploration-exploitation accounts, or of valence, which is often considered the key player in studies on flexibility and persistence, or both. Even though we did not directly assess physiological arousal in the current study, our findings suggest that both arousal and valence differently and uniquely contribute to the tendency to explore or exploit. If we speculate arousal is indicative of the noradrenergic system (Aston-Jones & Cohen, 2005), whereas valence may reflect changes in the dopaminergic system (Goschke & Bolte, 2014), our findings would be consistent with Cohen et al. (2007), who speculated that both systems might be involved in regulating control biases. This proposition fits well with increasing evidence from neurochemical and physiological studies suggesting interesting interactions between NE and DA and their sources (i.e., locus coeruleus and the ventral tegmental area) in regulating various kinds of processes (Ranjbar-Slamloo & Fazlali, 2020). However, as the functional role of these interactions still remains to be better understood, and the current study does not provide data to substantiate direct inferences on the involvement of neuromodulators, future work should focus on filling these gaps by including direct manipulations of catecholaminergic activity (Buffalari & Grace, 2007; Frank et al., 2009; Frank & Fossella, 2011; Kayser et al., 2015). This is particularly advised when considering the results presented here: a modulation of foraging behavior was only observed when accounting for participants’ performance pre-mood induction, an observation that emphasizes the importance of studying inter- and intra-individual differences in control processes (Mekern et al., 2019).

When interpreting our results, one important caveat has to be addressed. Specifically, although our experimental paradigm enabled us to examine the potential role of both arousal and valence in modulating the exploration-exploitation balance, mood induction protocols traditionally incorporate four conditions, reflecting excited (high arousal, high valence), sad (low arousal, low valence), anxious (high arousal, low valence), and calm (low arousal, high valence) induction conditions (Van Steenbergen et al., 2010). Because our main rationale to use the mood induction paradigm was to maximize the variance of mood impact on the exploration-exploitation trade-off, our protocol merely incorporated those two (out of four) unique dimensions that most likely resulted in the largest variability in arousal and valence scores. This might have resulted in an underestimation of the distinct contributions of, and interactions between, arousal and valence. However, even though the observed effects call for a more systematic investigation of the contributions of both NE and DA in regulating both the exploration-exploitation and persistence-flexibility balance, this does not render the current results any less interesting.

Concluding, our study shows that mood plays some role in the level of exploration-exploitation during foraging behavior, but that the directions of effects do not directly map onto the mood effects observed for modulation of flexible-persistent control tendencies. Together, this provides first insights into how processes related to arousal and valence differentially modulate foraging behavior. Considering links between mood and catecholaminergic neural mechanisms, our results might suggest a role of both noradrenaline and dopamine in regulating the exploration/exploitation trade-off, and signify that the relationship between exploration-exploitation and flexibility-persistence might be more complicated than previously assumed.
